# Intravascular immune cell labeling as a critical methodological tool for characterizing CD4^+^ T cells in the lung in EAE

**DOI:** 10.3389/fimmu.2026.1950269

**Published:** 2026-09-09

**Authors:** Gholamreza Azizi, Abdolmohamad Rostami

**Affiliations:** Department of Neurology, Thomas Jefferson University, Philadelphia, PA, United States

**Keywords:** CD4^+^ T cells, experimental autoimmune encephalomyelitis, intravascular CD45 labeling, lung-brain axis, neuroinflammation, tissue-resident immunity

## Abstract

**Introduction:**

Growing evidence highlights the lung -brain axis as a key contributor to CNS autoimmunity. However, accurate characterization of lung immune cells remains technically challenging because standard transcardial perfusion does not eliminate the lung’s extensive marginated intravascular leukocyte pool, allowing circulating cells to contaminate analyses of the lung parenchyma.

**Methods:**

Experimental autoimmune encephalomyelitis (EAE) was induced in mice by immunization with MOG_35_-_55_ peptide in complete Freund’s adjuvant, followed by pertussis toxin administration. Lung mononuclear cells were isolated after intravascular (IV) CD45 labeling, transcardial perfusion, and enzymatic digestion. Immune cell populations and intracellular cytokine production were analyzed by multicolor flow cytometry following ex vivo stimulation and intracellular staining.

**Results:**

We performed *in vivo* CD45 labeling by IV injection of a fluorochrome-conjugated anti-CD45 antibody immediately before euthanasia. Despite extensive transcardial perfusion, 73–85% of isolated lung leukocytes were IV-labeled, demonstrating substantial persistence of IV cells. In contrast, true lung parenchymal (IV^-^) CD4^+^ T cells from both naïve and EAE mice exhibited a markedly enriched effector-memory phenotype, elevated expression of tissue residency-associated markers (CD69 and CD11a), and significantly greater production of IL-17, GM-CSF, and IFN-γ than circulating (IV^+^) cells. Notably, IV^+^ pulmonary vascular CD4^+^ T cells closely resembled peripheral blood CD4^+^ T cells but exhibited moderately increased expression of selected cytokines and transcription factors.

**Discussion:**

These findings demonstrate that transcardial perfusion alone is insufficient to accurately distinguish lung parenchymal from intravascular immune cells. We propose that *in vivo* intravascular labeling should be incorporated as a methodological standard for studies investigating pulmonary immune responses.

## 1 Introduction

The central nervous system (CNS) has historically been considered an immune-privileged site; however, pathogenic T cell infiltration remains a hallmark of neuroinflammatory diseases such as multiple sclerosis (MS) and its animal model, experimental autoimmune encephalomyelitis (EAE) (1). Emerging evidence supports a “Hub-and-Spoke” paradigm of CNS autoimmunity, in which effector CD4^+^ T cells undergo licensing, metabolic adaptation, and transcriptional reprogramming within peripheral non-lymphoid organs, particularly the lung, before migrating to the CNS (2–7). During neuroinflammation, autoreactive T cells accumulate in the pulmonary microenvironment and acquire pathogenic effector memory phenotypes, including IL-17– and GM-CSF–producing subsets. Consistent with this, increased frequencies of MOG-specific effector-memory CD4^+^ T cells have been detected in the lungs of EAE mice (7–9). However, accurately defining T cell dynamics within the highly vascularized pulmonary architecture remains a major methodological bottleneck. Conventional transcardiac perfusion and tissue dissociation cannot reliably distinguish true parenchymal (Par) immune cells from the extensive marginated intravascular (IV) leukocyte pool. Indeed, standard perfusion alone can leave approximately 74%–97% of recovered lung leukocytes within the pulmonary vasculature (10–13). While IV antibody labeling is widely recognized in mucosal immunology, studies investigating the “lung-brain axis” in EAE continue to rely almost exclusively on transcardiac perfusion alone (3, 4). Consequently, incorporating IV-CD45 staining is indispensable to prevent the misattribution of marginated vascular cells to the lung parenchyma and to accurately define the phenotype, localization, and functional behavior of pulmonary T cells during EAE. While baseline immune kinetic differences between naive and EAE lungs are well documented (3, 4, 8) this study specifically evaluates the methodological necessity of *in vivo* intravascular labeling to distinguish true pulmonary parenchymal CD4^+^ T-cell responses from marginated vascular contaminants during neuroinflammation.

## 2 Materials and methods

### 2.1 Animals and experimental design

Male and female C57BL/6 mice (3 months old) were housed under specific pathogen-free conditions with ad libitum access to food and water. For each experiment, age- and sex-matched mice were used with an equal sex distribution (n = 4 mice per group per experiment; 2 males and 2 females). All experiments were performed in two independent biological replicates, and the data presented in the figures are from one representative experiment (n = 4), unless otherwise indicated. Animals were randomly assigned to experimental groups before immunization or tissue collection. All animal procedures were conducted in accordance with institutional guidelines and approved by the Institutional Animal Care and Use Committee (IACUC).

### 2.2 EAE induction protocol

EAE was induced as previously described (14). In brief, mice received a subcutaneous injection of 200 µg MOG_35-55_ peptide (GenScript, CA, USA) emulsified in Complete Freund’s Adjuvant (Thermo Scientific). Additionally, 200 ng of pertussis toxin (Sigma-Aldrich) was administered intraperitoneally on days 0 and 2 post-immunization. Clinical signs were evaluated daily using the following scoring system: 0, no symptoms; 1, tail paralysis; 2, single hindlimb paralysis; 3, paralysis of both hindlimbs; 4, abdominal paralysis; 5, death.

### 2.3 Mononuclear cell isolation

Mice were anesthetized with a ketamine-xylazine mixture. Intravascular immune cells were labeled by intravenous injection of 2 µg of BV421-conjugated anti-CD45 antibody (clone 30-F11) 3 minutes before transcardial perfusion with cold PBS. Clone 30-F11 does not appreciably interfere with subsequent ex vivo staining with Alexa Fluor 700 (AF700)-conjugated anti-CD45 antibody (clone 13/2.3), as validated in peripheral blood where all immune cells were double-positive for both antibodies (**Supplementary Figure 1A**). The lungs were harvested and enzymatically digested with Liberase (200 µg/mL; Sigma-Aldrich) and DNase (50 µg/mL; Stem Cell) in RPMI for 30 minutes at 37 °C, followed by mechanical dissociation. Lung debris was eliminated using the Debris Removal Solution (Miltenyi Biotec).

### 2.4 Flow cytometry, intracellular staining, and gating strategy

For immunophenotyping, single-cell suspensions were first incubated with an Fc receptor-blocking reagent and subsequently stained with fluorophore-conjugated antibodies against surface markers (**Supplementary Table 1**), followed by viability staining with 7-AAD (Biolegend). For intracellular cytokine staining, cells were stimulated with PMA (50 ng/mL; Sigma-Aldrich), ionomycin (500 ng/mL; Sigma-Aldrich), and GolgiPlug (1 µg/mL; BD Biosciences) at 37 °C for 5 h. Following stimulation, single-cell suspensions were first incubated with Fixable Viability Stain (Zombie Yellow, Biolegend) prior to surface staining. Then cells were stained for surface markers, fixed, and permeabilized using Caltag Fix/Perm reagents (Invitrogen) according to the manufacturer’s instructions, followed by staining with fluorophore-conjugated antibodies against intracellular cytokines and transcription factors (**Supplementary Table 1**).

Data were acquired using a FACSAria Fusion II flow cytometer (BD Biosciences) and analyzed with FlowJo software (version 10.8; Tree Star). Sequential doublet discrimination was performed using FSC-H versus FSC-A and SSC-H versus SSC-A parameters. Gating boundaries, non-specific binding, and background fluorescence were established using fluorescence-minus-one (FMO) controls, isotype control, and unstained and unstimulated samples. A complete list of monoclonal antibodies, including target antigens, clones, fluorophores, and manufacturers, is provided in **Supplementary Table 1**. Parenchymal (IV^-^) CD4^+^ T cells were identified as exhibiting a tissue-resident memory (TRM) phenotype based on the elevated expression of CD69 and CD11a, consistent with previously established phenotypic characteristics of pulmonary CD4^+^ TRM populations (15, 16).

### 2.5 Statistical analysis

Statistical analyses were performed using GraphPad Prism (v8.0, GraphPad Software). Data normality and variance homogeneity were assessed using the Shapiro-Wilk test and Levene’s test, respectively. For multi-group comparisons, a one-way analysis of variance (ANOVA) was performed, followed by Tukey’s *post hoc* test for multiple comparisons. All data represent biological replicates from individual animals (n), with data presented as mean ± SEM. A *p-value* ≤ 0.05 was considered statistically significant.

## 3 Results

**Figure 1:**
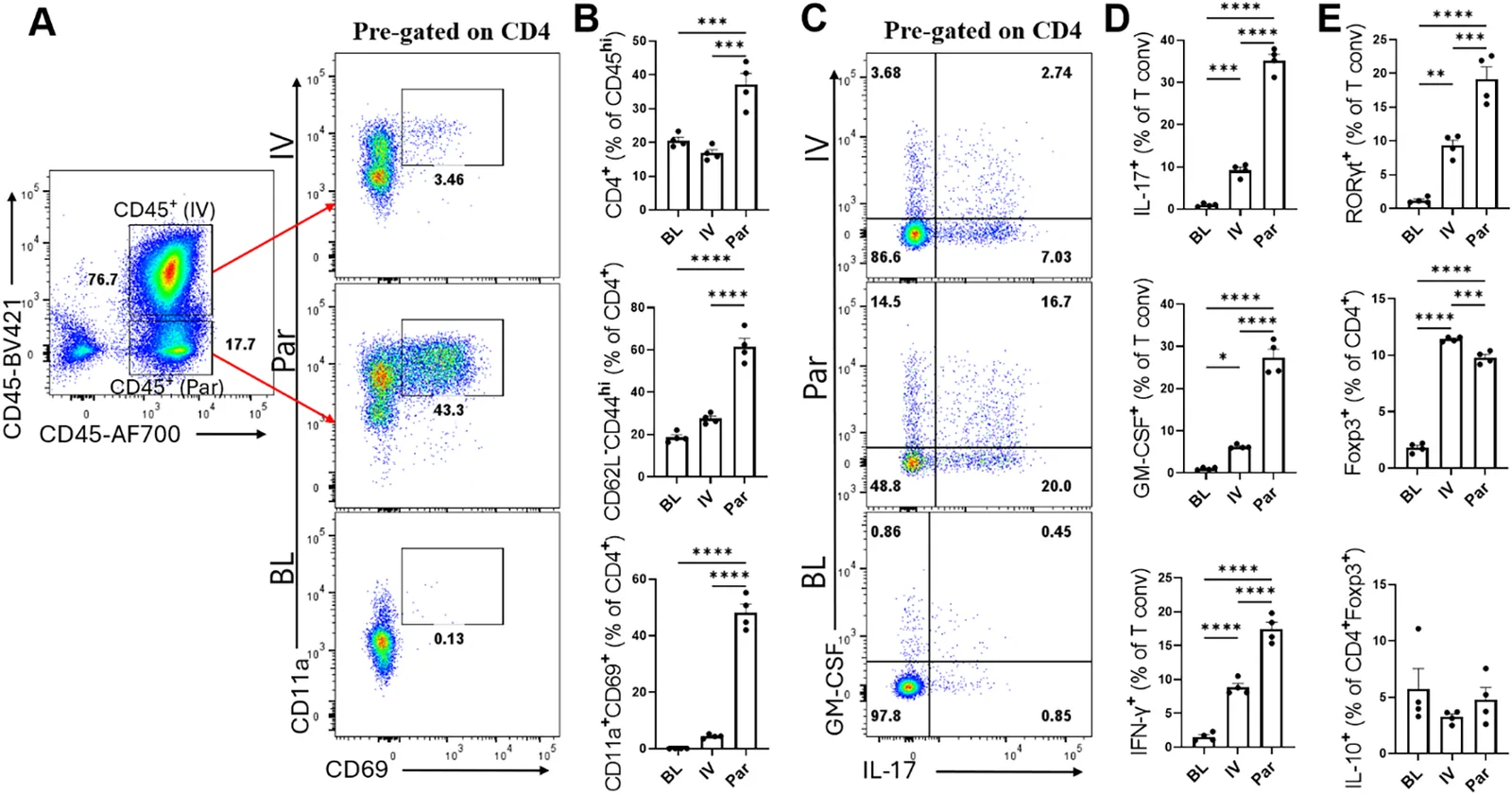
Phenotypic characterization of lung parenchymal (Par) and intravascular (IV) CD4^+^ T cells in EAE. Three-month-old male and female C57BL/6 mice were immunized to induce EAE (total n = 8; equal sex distribution, 4 males and 4 females). At the peak of clinical severity, circulating immune cells were labeled *in vivo* by intravenous injection of BV421-conjugated anti-CD45 antibody 3 min before euthanasia. We collected blood via cardiac puncture, perfused mice, enzymatically digested lung tissue, and isolated MNCs. Cells were either analyzed directly for surface marker expression or stimulated with PMA/ionomycin to assess intracellular cytokine production. **(A)** Representative flow cytometry plot showing *in vivo* labeling with intravascular (IV) BV421-conjugated anti-CD45 antibody and *ex vivo* staining with AF700-conjugated anti-CD45 antibody. We identified cells positive for both antibodies as IV cells, whereas cells stained only with anti-CD45-AF700 were considered parenchymal (Par) cells. **(B)** CD4^+^ T cell frequency and expression of CD69, CD11a, CD62L, and CD44. **(C–E)** Following stimulation, conventional (Foxp3^-^) CD4^+^ T cells were analyzed for intracellular expression of IL-17, GM-CSF, IFN-γ, and RORγt, while Foxp3^+^ CD4^+^ T cells were evaluated for their frequency and IL-10 expression. Data are representative of two independent experiments with similar results (n = 4 mice/group/experiment; age- and sex-matched). Mice were randomly assigned to experimental groups. Statistical analysis was performed using one-way ANOVA, followed by Tukey’s *post hoc* test *P < 0.05; **P < 0.01; ***P < 0.001; ****P < 0.0001.

Building upon the previous observations, we performed control experiments in naïve and EAE mice using *in vivo* IV-CD45 labeling as a standard method (10, 12, 15, 17). To distinguish parenchymal from IV cells, mice with peak EAE at 20 days post-immunization (**Supplementary Figure 1B**) were intravenously injected with a BV421-labeled anti-CD45 antibody (Ab) shortly before euthanasia. Following extensive transcardiac perfusion, IV CD45^+^ cells accounted for 73-85% of the total isolated mononuclear cells, with this range representing the minimum–maximum observed among individual mice (n = 8). These findings indicate that, under our experimental conditions, a substantial proportion of cells within the isolated lung cell population remained within the pulmonary vasculature despite extensive transcardiac perfusion. Moreover, labeled (IV^+^) and unlabeled (true parenchymal) lung CD4^+^ T-cell populations exhibited markedly distinct phenotypes, with IV cells somewhat resembling peripheral blood CD4^+^ T-cell phenotypes (**Figure 1**). Subsequent analyses revealed that parenchymal lung mononuclear cells (MNCs) in EAE mice harbored a higher frequency of CD4^+^ T cells than both IV-labeled cells and peripheral blood cells. Specifically, these parenchymal CD4^+^ T cells displayed a significantly higher frequency of the effector-memory (E.M.) phenotype and tissue-resident markers (CD69 and CD11a) (15) than in the two distinct circulating compartments (**Figures 1A, B**). Furthermore, more parenchymal conventional (Foxp3^-^) CD4^+^ T cells produced IL-17, GM-CSF, and IFN-γ (**Figures 1C, D**). Although RORγt expression by conventional CD4^+^ T cells mirrored cytokine expression, the frequency of Foxp3^+^ CD4^+^ T cells was significantly higher in the IV-labeled compartment than in the parenchymal and peripheral blood compartments. However, their capacity to produce IL-10 was not significantly altered (**Figure 1E**). Our results demonstrated that while parenchymal CD4^+^ T cells differed significantly from both IV and peripheral blood compartments; however, the IV-labeled CD4^+^ T cells and peripheral blood populations were not entirely identical. Notably, IV-labeled CD4^+^ T cells exhibited increased cytokine expression, along with higher levels of the lineage-defining transcription factors RORγt and Foxp3, compared with their peripheral blood counterparts. This phenotypic divergence suggests that the pulmonary IV compartment features a slightly higher proportion of marginated effector cells (11, 18, 19), rather than simply representing a passive extension of the systemic circulation.

**Figure 2:**
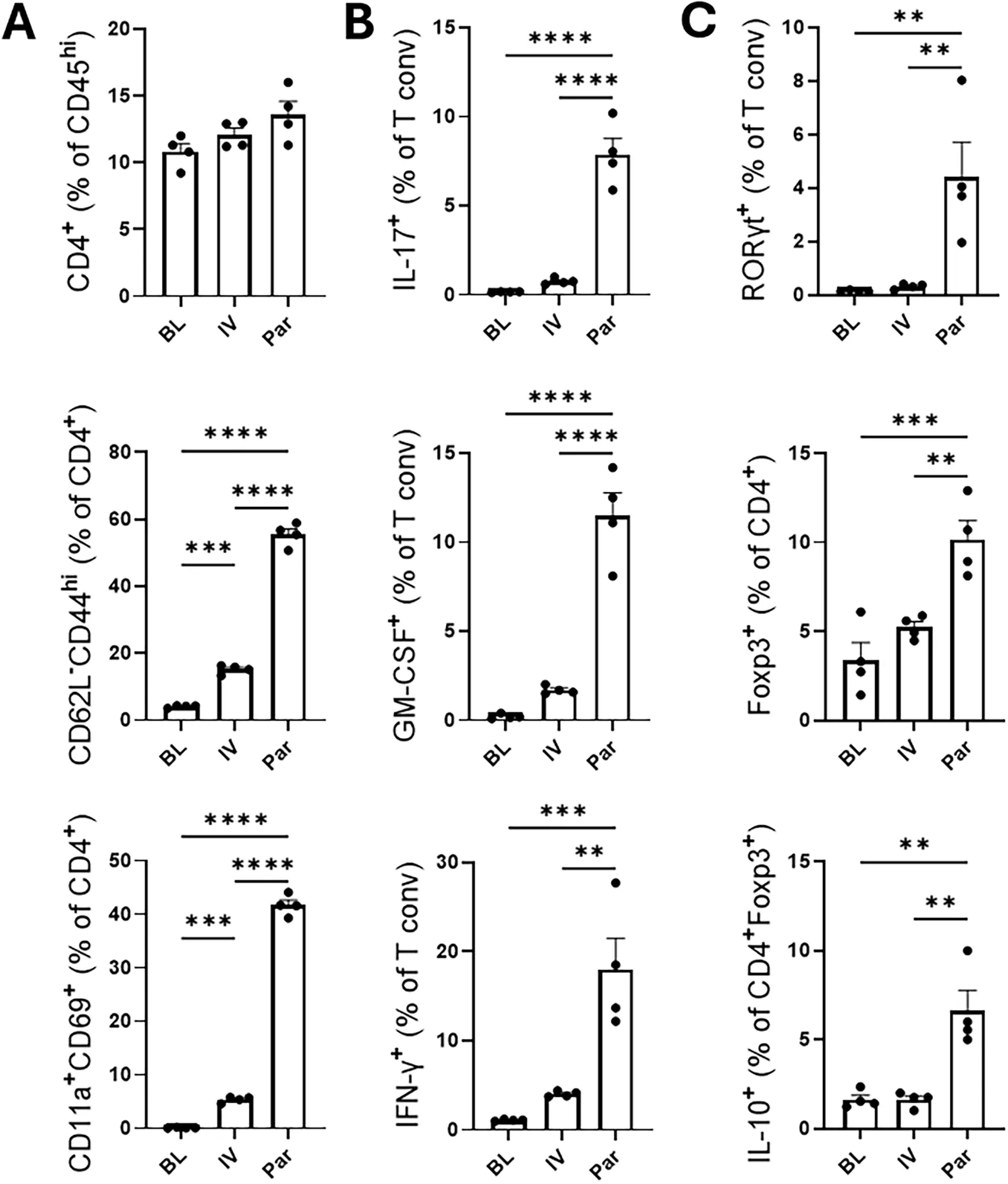
Phenotypic characterization of lung parenchymal (Par) and intravascular (IV) CD4^+^ T cells in naïve mice. Circulating CD45^+^ cells were labeled *in vivo* by intravenous injection of BV421-conjugated anti-CD45 antibody 3 min before euthanasia in 3-month-old male and female C57BL/6 mice (total n = 8; equal sex distribution, 4 males and 4 females). We collected blood via cardiac puncture, perfused the mice, and isolated lung MNCs. Cells were either analyzed directly for surface marker expression or stimulated with PMA/ionomycin to assess intracellular cytokine production. **(A)** Expression of CD69, CD11a, CD62L, and CD44 on CD4^+^ T cells. **(B, C)** Following stimulation, CD4^+^ T cells were analyzed for intracellular expression of IL-17, GM-CSF, IFN-γ, IL-10, Foxp3, and RORγt. Data are representative of two independent experiments with similar results (n = 4 mice/group/experiment; age- and sex-matched). Mice were randomly assigned to experimental groups. Statistical analysis was performed using one-way ANOVA followed by Tukey’s *post-hoc* test. **P < 0.01; ***P < 0.001; ****P < 0.0001.

Because pulmonary inflammation during EAE can alter leukocyte distribution and activation states, we investigated whether similar CD4^+^ T-cell compartmentalization and phenotypic heterogeneity were also present under steady-state conditions. While the overall frequency of CD4^+^ T cells among isolated MNCs did not differ significantly between the peripheral blood, intravascular, and parenchymal compartments, their phenotypic profiles closely mirrored those observed in EAE mice. In naïve animals, parenchymal CD4^+^ T cells exhibited significantly higher expression of CD69 and CD11a, together with a more pronounced E.M. phenotype, compared with both IV and peripheral blood CD4^+^ T cells (**Figure 2A**). Following ex vivo stimulation, parenchymal conventional CD4^+^ T cells displayed elevated expression of IL-17, GM-CSF, IFN-γ, and RORγt (**Figures 2B, C**), while the parenchymal regulatory compartment contained a higher frequency of Foxp3^+^CD4^+^ T cells and exhibited increased IL-10 expression (**Figure 2C**). Consistent with our findings in EAE mice, IV-labeled CD4^+^ T cells in naïve mice displayed an intermediate phenotype, exhibiting functional and transcriptional characteristics that more closely resembled peripheral blood CD4^+^ T cells while retaining limited similarity to the parenchymal compartment.

## 4 Discussion

Although transcardial perfusion is widely utilized to clear blood-borne cells from the lungs, our findings are in alignment with previous reports (12, 13, 20) demonstrate that hydrostatic flushing alone is insufficient to eliminate IV contamination. Residual vascular leukocytes frequently persist following standard transcardial perfusion due to anatomical short-circuiting of the pulmonary loop, physical trapping within collapsed alveolar capillaries, and the firm adherence of marginated immune cells to the vascular endothelium. To accurately distinguish bona fide parenchymal cells from these remaining IV leukocytes, we employed an *in vivo* IV labeling approach using a fluorophore-conjugated anti-CD45 Ab administered before euthanasia and tissue harvest. Using this strategy, we identified marked phenotypic and functional differences between circulating and parenchymal CD4^+^ T-cell populations in both naïve and EAE mice. Parenchymal CD4^+^ T cells were enriched for an E.M. phenotype, expressed significantly higher levels of tissue-residency markers, and exhibited substantially greater effector function than their circulating counterparts upon *ex vivo* stimulation. Ultimately, failing to account for these resilient IV contaminants can severely confound analyses of lung-localized immunity, a factor that is especially critical when interpreting lung CD4^+^ T-cell involvement within the context of the Lung-Brain axis.

IV-labeled CD4^+^ T cells exhibited a phenotype that more closely resembled the peripheral blood compartment, while showing only limited similarity to the parenchymal compartment. This intermediate profile likely reflects leukocyte margination (10) within the unique architecture of the pulmonary capillary bed. The low-pressure microvascular network of the lung acts as a physiological reservoir that transiently retains activated lymphocytes due to reduced deformability and upregulated adhesion molecules (21). Collectively, these findings highlight that analysis of lung-isolated immune cells following standard perfusion alone provides an incomplete representation of the dynamic pulmonary immune landscape, underscoring the necessity of *in vivo* IV labeling to accurately distinguish IV from parenchymal compartments.

Here, we acknowledge a potential limitation related to EAE-associated vascular permeability and inflammation that could influence the efficiency of IV antibody labeling. In our experimental protocol, BV421-conjugated anti-CD45 antibody was administered intravenously and allowed to circulate for 3 min before tissue collection and perfusion. This short labeling interval was designed to preferentially label leukocytes exposed to the vascular lumen while minimizing the potential for antibody extravasation into the tissue parenchyma. Importantly, flow cytometric analysis of lung leukocytes from EAE mice consistently demonstrated clear separation between IV^+^ and IV^−^ populations, without an apparent broad intermediate staining pattern suggestive of substantial nonspecific antibody diffusion. Furthermore, CD69 and CD11a high expression was predominantly observed within the IV^−^ CD4^+^ T-cell population, consistent with the expected enrichment of tissue-resident or tissue-retained cells within the parenchymal compartment. Nevertheless, we acknowledge that EAE-associated vascular inflammation may increase vascular permeability and that complete exclusion of limited antibody extravasation cannot be established solely by flow cytometric analysis.

## Data Availability

The original contributions presented in the study are included in the article/**Supplementary Material**. Further inquiries can be directed to the corresponding author.

## Supplementary material

Supplementary Table 1 Anti-mouse antibodies used for flow cytometry.

Supplementary Figure 1 Intravascular labeling of peripheral blood leukocytes and clinical course of EAE. **(A)** Representative flow cytometry plot showing IV labeling of peripheral blood leukocytes with BV421-conjugated anti-CD45 antibody, followed by ex vivo staining with AF700-conjugated anti-CD45 to identify accurate labeling. **(B)** Clinical EAE scores over time, showing the onset and progression of EAE following immunization. Data are representative of a 2 independent experiment (total n = 8).
