# Supplementary material for: Intravascular immune cell labeling as a critical methodological tool for characterizing CD4+ T cells in the lung in EAE
Source: Front Immunol. 2026 Sep 9;17:1950269. doi: 10.3389/fimmu.2026.1950269 (PMC13597352; doi:10.3389/fimmu.2026.1950269)
Supplement: Supplementary file 1 [file DataSheet1.docx]

**Table S1. Anti-mouse Antibodies Used for Flow Cytometry.**

| **Antigen** | **Fluorochrome** | **Clone** | **Supplier** | **Category** |
| --- | --- | --- | --- | --- |
| CD4 | BV650 | GK1.5 | Biolegend | Surface |
| CD4 | BV605 | RM4-4 | Biolegend | Surface |
| CD44 | PE/Cy7 | IM7 | Biolegend | Surface |
| CD44 | PE | IM7 | Biolegend | Surface |
| CD45 | AF700 | I3/2.3 | Biolegend | Surface |
| CD45 | BV421 | 30-F11 | Biolegend | Surface |
| CD69 | PE/Dazzle™ 594 | H1.2F3 | Biolegend | Surface |
| CD11a | PE/Cy7 | 2D7 | Biolegend | Surface |
| CD62L | BV785 | MEL-14 | Biolegend | Surface |
| CD62L | APC | MEL-14 | Biolegend | Surface |
| IFN-γ | APC/Fire750 | W18272D | Biolegend | Intercellular |
| GM-CSF | PE/Dazzle™ 594 | MP1-22E9 | Biolegend | Intercellular |
| IL-10 | PE/Cy7 | JES5-16E3 | Biolegend | Intercellular |
| IL-17 | BV785 | TC11-18H10.1 | Biolegend | Intercellular |
| Foxp3 | PE | FJK-16S | eBioscience | Intercellular |
| RORγt | APC | AFKJS-9 | eBioscience | Intercellular |
| CD; Cluster of Differentiation, IL; Interleukin, IFN; Interferon, GM-CSF; Granulocyte-macrophage colony-stimulating factor, Foxp3; Forkhead box P3, RORγt: Retinoic acid receptor-related orphan receptor gamma t | | | | |

**Figure S1. Intravascular labeling of peripheral blood leukocytes and clinical course of EAE.** (**A**) Representative flow cytometry plot showing IV labeling of peripheral blood leukocytes with BV421-conjugated anti-CD45 antibody, followed by ex vivo staining with AF700-conjugated anti-CD45 to identify accurate labeling. (**B**) Clinical EAE scores over time, showing the onset and progression of EAE following immunization. Data are representative of a 2 independent experiment (total n = 8).
